# Gastrointestinal functioning and menstrual cycle phase in emerging young adult women: a cross-sectional study

**DOI:** 10.1186/s12876-023-03036-3

**Published:** 2023-11-21

**Authors:** Sivanne Mendelson, Preethashree Anbukkarasu, Jeffrey E. Cassisi, Widaad Zaman

**Affiliations:** 1 Department of Psychology, University of Central Florida, Orlando, FL 32816 USA; 2 College of Medicine, University of Central Florida, 6850 Lake Nona Blvd, Orlando, FL 32827 USA

**Keywords:** DGBI, FGID, GI symptoms, Menstrual cycle, Menses, Health anxiety

## Abstract

**Background:**

Women experience more severe gastrointestinal (GI) symptoms compared to men. The onset of puberty and the menstrual cycle may influence these differences. Additionally, health anxiety is an important construct that has been shown to play a role in increased symptomatology across many medical conditions. Using standardized clinical measures often employed to assess disorders of gut-brain interaction (DGBI) we aimed to identify differences of GI functioning across menstrual cycle phases and to evaluate the role of health anxiety in this relationship.

**Methods:**

Six hundred three participants completed a survey including functional GI assessment scales (PROMIS-GI®), an abdominal pain scale and map, and a health anxiety measure. They were grouped by menstrual cycle phases (Menses, Follicular, Early-Luteal, and Premenstrual) based on self-reported start date of most recent period. Multivariate analyses of covariance were conducted to identify differences between menstrual cycle phase and scores on the symptom scales. Heath anxiety was included as a covariate in all analyses.

**Results:**

No significant differences were found between menstrual cycle group and PROMIS-GI scores. Higher GI-symptom and pain levels were found as health anxiety increased. Pain in the hypogastric region of the abdomen was significantly higher during the Menses phase when compared to Early-Luteal and Premenstrual phases. A subset of participants with DGBI diagnoses demonstrated significantly higher GI-symptom severity on several PROMIS-GI scales when compared to matched controls who did not have those diagnoses. In addition, participants with DGBI diagnoses reported significantly greater pain across multiple abdominal regions than their non-diagnosed counterparts.

**Conclusions:**

GI symptom levels as measured by the PROMIS-GI scales in otherwise healthy women were not dependent on menstrual cycle phase. Yet, the PROMIS-GI scales were sensitive to symptom differences in women with DGBI diagnoses. Overall, this study demonstrated that the PROMIS-GI measures are unlikely to be affected by gynecological functioning in healthy young women. We argue that the abdominal pain map is an essential addition to classification and diagnosis.

## Background

Evidence for sex differences in gastrointestinal (GI) functioning exist across many studies. For example, women reported significantly more GI symptoms, such as abdominal pain and bloating compared to men in two studies [1, 2]. In another study using a community sample of patients with irritable bowel syndrome (IBS), women reported more constipation, while men reported more diarrhea [3]. Additionally, two studies found higher levels of GI symptoms (e.g., abdominal pain, bloating, and cramping) in women [1, 4]. In yet another, using matched samples of women and men with equal levels of IBS severity, chronicity, and psychological distress, women had greater extraintestinal symptoms, such as chronic pain and headaches, compared to men [5]. Lastly, prevalence rates of disorders of gut-brain interaction (DGBI) are higher in women compared to men [1, 6]. IBS, one of the most prevalent DGBI, occurs in twice as many women than men [7]. Interestingly, GI disorders occur with similar prevalence across biological sex in prepubescence [8]. It is possible, therefore, that biological sex differences in GI symptom prevalence among adults may be associated with the changes that occur during puberty and the emergence of the menstrual cycle. This too is consistent with the large literature on hormonal influences on clinical and experimental pain [9].

The relationship between specific GI symptoms and the menstrual cycle has been studied to some extent. A study on women with inflammatory bowel disease (IBD) found that perimenstrual GI symptoms were common in both healthy women and women with IBD, indicating that symptoms vary with menstrual cycle phase regardless of diagnosis [10]. However, the same authors found evidence to the contrary in another study [11]. Menstrual cycle phase was found to influence stool consistency in women. Those with dysmenorrhea reported significantly more nausea and decreased food intake than nondysmenorrheic individuals [12]. In women with IBS, symptomatology including rectal sensitivity was considerably exacerbated during menses [13]. In a sample of healthy women taking oral contraceptives, stool consistency and frequency, abdominal pain, reflux, and indigestion showed significant variations based on menstrual cycle timepoint [14]. Overall, the association between GI disorders and the menstrual cycle have been studied in the context of bowel-related disorders and dysmenorrhea. More research is needed to identify a comprehensive and standardized list of GI symptoms across the phases of the menstrual cycle and to clarify the relationship between the two at the community level. Ultimately, assessment of DGBI may be more accurate with consideration of the menstrual cycle and other hormonal influences on GI functioning.

### The menstrual cycle

At the broadest level, the menstrual cycle is made up of the follicular and luteal phases [15, 16]. The average length of a menstrual cycle is 28 days, with most cycles ranging from 22 to 36 days [17]. Variations in the operational definition of the phases can be seen across studies; Namely, with the inclusion or exclusion of menses in the follicular phase and the premenstrual phase in the luteal phase. The follicular phase typically ranges between 7–22 days, with an average duration of 14 days. It begins on the first day of menstruation and ends with ovulation [18]. Menstruation, or menses, is the discharge of blood from the uterine lining on a cyclical basis, and is regulated by hormone levels including estrogen, progesterone, and luteinizing hormone. Menses is estimated to last between 3–7 days in healthy women, and bleeding lasting more than 7 days is considered a menstrual abnormality [18]. Various studies have reported the luteal phase on a 14 –16-day range [13, 17, 18]. Thus, there has been significant variation in operationally defining each phase of the menstrual cycle across studies.

Fluctuations of pain, cognitive, affective, and physical symptoms according to the phases of the menstrual cycle have been described across studies [9, 19, 20]. However, findings regarding the specific patterns of symptom changes through the phases of the menstrual cycle are inconclusive. In general, the premenstrual phase is associated with increased somatic symptoms, fluid retention, negative affect, weight gain, depression, painful breasts, and lower overall health. During menses, somatic symptoms, pain, autonomic reaction, and fluid retention increase significantly [19–21]. Furthermore, the follicular phase is associated with lower pain [9].

### Health anxiety

The American Psychiatric Association defines health anxiety as excessive fear of serious illness and misinterpretation of physical symptoms or bodily changes [22]. Health anxiety, previously called hypochondriasis, has been associated with symptom complaints in many different body systems including cardiovascular, somatosensory, head and neck, and gastrointestinal [23]. Higher levels of psychological distress and global health anxiety in females have been suggested [24], yet there are few studies that examine the effects of health anxiety across the menstrual cycle. One study found increases in health anxiety and stress during the luteal phase [25]. The role of health anxiety on the menstrual cycle is crucial to the current study given its high comorbidity rates with GI symptoms and DGBI [26–29]. This is further illustrated by one cross-sectional study with two groups of women either in the late-luteal or the follicular phase of the menstrual cycle. They found that high levels of health anxiety increased perceived stress and symptom severity primarily in the premenstrual phase [25]. This was the only study, to our knowledge, that examined health anxiety across the different phases of the menstrual cycle. Unfortunately, the sample size of 38 women was too small to produce generalizable results and it did not include measures of GI functioning. Thus the relationship between GI functioning, health anxiety, and the menstrual cycle requires further investigation.

### Objectives of the current study

The primary objective of this study is to examine the relationship between GI symptoms experienced by emerging healthy young adults during different phases of the menstrual cycle using standardized clinical patient report measures. Challenges to studying the menstrual cycle in a large population are the limitations of participant accuracy with self-report and recall. Without direct physiological measures, placement of an individual within their cycle requires recall and self-report of the start date of their most recent period, via the calendar method [30]. Given this limitation and the variation in the literature operationally defining the menstrual phases, the naming approaches of Draper et al. (2018) were followed [31]. We operationally defined the phases of menstrual cycle as follows: Menses is the 7-day interval starting with the first day of the period, and the Follicular phase is the 7-day interval following directly after. The Early-Luteal phase is the 7-day interval following the end of the follicular phase, and the Premenstrual phase is the last 7-day interval of the luteal phase. Any research based on large populations will inevitably contain error in categorizing some participants and we chose a parsimonious approach to placing women into these four subgroups.

A secondary objective of this study is to provide insight into the psychological factors that may influence the self-report of GI symptoms in women, by exploring the relationship between health anxiety and GI symptoms across the menstrual cycle.

The tertiary objective is to evaluate whether the Patient-Reported Outcomes Measurement Information System (PROMIS®) GI scales are affected by menstrual phase. These standardized GI measures offer a method of tracking symptoms in the medical setting [32], and we have previously extended their use to community samples [33]. While the literature indicates that GI-related symptom variations may occur across the menstrual cycle, no previous studies were found using the PROMIS-GI measures to examine this relationship. It is important for GI specialists to understand the degree to which patient reporting on the PROMIS-GI scales is influenced by other systems, including the female reproductive system.

### Hypotheses

Upon review of the literature, we hypothesized that there would be higher GI symptoms as measured by the PROMIS-GI scales during the luteal phases of the menstrual cycle compared to the follicular phase. Specifically, we predict that women in the premenstrual phase will demonstrate higher GI and abdominal pain symptoms that would be reflected in their PROMIS-GI score [11].

**Fig. 1:**
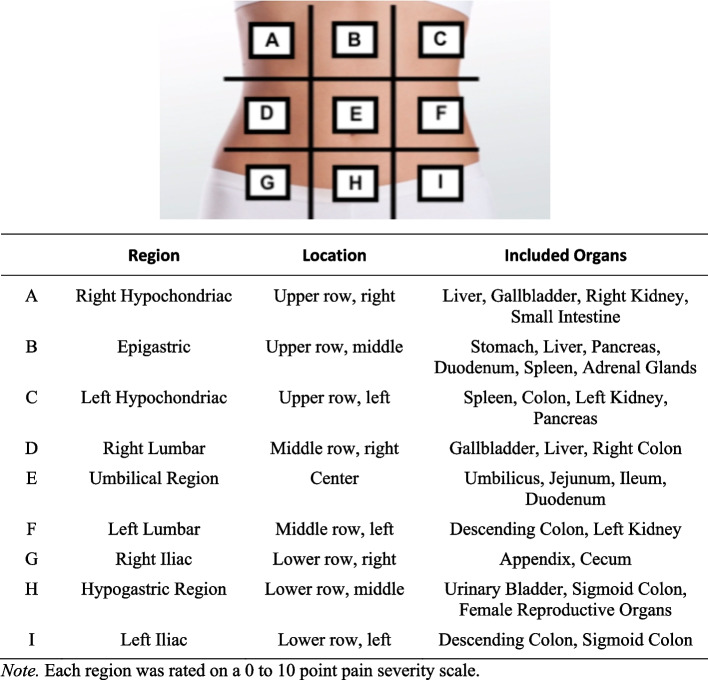
Abdominal pain map with 9 clinical subdivisions of abdominal region

There is a need to more precisely characterize abdominal pain related to GI and menstrual symptoms. To achieve this, we utilized an abdominal pain map, illustrated in Fig. 1. We hypothesized that pain reported during the menses and luteal phases would be higher in the lower abdominal region, where the female reproductive organs are located, and in line with literature on gynecologic disorders [34–36].

Health anxiety has been repeatedly shown to affect symptom severity across medical conditions. Use of this measure is exploratory and as such directional hypotheses are not offered. Research by Ross et al. has demonstrated that psychological distress in the form of health anxiety is highly predictive of GI health [37, 38]. We therefore hypothesized that higher levels of health anxiety would be associated with higher GI symptom severity and increased intensity of symptoms throughout all phases of the menstrual cycle.

## Methods

### Participants

Participants were English speaking female undergraduates between the ages of 18 and 25 who were enrolled in introductory psychology courses at a large, public university in the United States. Introductory psychology is a required course for all programs at this university, which ensures that a diverse range of majors and backgrounds are represented in the undergraduate population sample. As part of the course requirement, students must participate in research activity and earn credit toward their undergraduate psychology courses. Prior to data collection, the study protocol was approved by the University of Central Florida IRB (STUDY00001918).

### Measures

Participants were recruited via SONA®, an online research recruitment application utilized by the university. They completed an online survey in Qualtrics^XM^ including demographic and medical history information, as well as specific questions assessing GI symptoms and health anxiety. Two validity check questions were included in the questionnaire as a determining variable for respondent data elimination or retention. The survey took approximately 30 minutes to complete.

#### Functional gastrointestinal assessment

The NIH PROMIS-GI symptom scales. The National Institutes of Health (NIH) developed the Patient Reported Outcomes Measurement Information System (PROMIS®) Gastrointestinal Symptom Scales (PROMIS-GI) in 2014. The PROMIS-GI scales have been validated as effective measures of a broad range of GI symptoms within the general and clinical populations and may be effective in identifying clinical thresholds for action [32, 39]. The PROMIS-GI scales have good psychometric properties and construct validity [32]. The PROMIS-GI version 1 scales evaluate eight GI symptom domains: Abdominal Pain (6 items), Gas/bloating (v1.1; 12 items), Diarrhea (5 items), Constipation (9 items), Bowel Incontinence (4 items), Gastroesophageal Reflux (13 items), Disrupted Swallowing (8 items), and Nausea/vomiting (4 items). All items ask participants to recall symptoms that occurred within the past 7 days. Scores were calculated using the PROMIS website (www.healthmeasures.net) and provided as a standardized T-score with a mean of 50 and a SD of 10. Higher T-scores indicate more severe symptoms compared to the national normative sample described in Spiegel et al. [32]. T-scores were converted into GI symptom severity levels using the suggested PROMIS T-Score range of mild (T-scores between 55 and 60), moderate (T-scores between 60 and 70), and severe (T-scores above 70).

#### Abdominal pain scale and map

The pain scale is an adaptation of the numeric rating scale that is commonly used in many medical specialties [40]. Individuals are asked to select the areas where they currently experience pain on a scale from 0 to 10, with 0 representing least pain and 10 representing the most pain possible. The abdomen was graphically divided into nine regions and individuals provided a numeric rating for each region. This was facilitated by a drawing of the abdomen with a 3 × 3 grid drawn on top of the image. Figure 1 provides the Abdominal Pain Map and a clinical description for each region.

#### Short Health Anxiety Inventory (SHAI)

The Health Anxiety Inventory was developed in 2002 to measure healthy anxiety and the cognitive factors associated with hypochondriasis independent of physical health status [41]. The original Health Anxiety Inventory has 64 items, with each item on a four-point Likert scale [42]. An abbreviated 18-item scale was also constructed, termed the Short Health Anxiety Inventory (SHAI) [42]. The SHAI is preferred in research and clinical settings because of its length and comparable psychometric properties to the original Health Anxiety Inventory, including reliability and convergent, divergent, predictive, construct, and criterion validity. The SHAI is comprised of three factors that assess the perceived likelihood of becoming ill, body vigilance, and the perceived severity of becoming ill with a recall period of 6 months [41]. The first 14 items on the SHAI represent the main section. Each item is scored on a 4-point scale of 0–3 and then totaled to create a final score. Higher scores indicate higher levels of health anxiety [42]. For this study, only the first 14 items of the SHAI were used.

#### Menstrual cycle and medical history items

Several items concerning medical history were included in the survey, including current contraceptive use, current pregnancy status, and whether participants were receiving medical treatment for dysmenorrhea, premenstrual syndrome (PMS), and/or amenorrhea. Participants also responded to a single item involving the date of the start of their last period. This information, in combination with the date of the response submission, was used to calculate the days since last period and determine menstrual phase group assignment.

### Procedure

The dataset was reviewed prior to data analysis and 731 individuals attempted the survey. After cleaning the data there were 603 total participants in this study: Individuals were eliminated from analyses if they reported irregular cycles (menses occurred greater than 35 days prior to survey completion), incorrectly responded to one or more validity check items, failed to complete the survey, or provided inconsistent responses for menses onset and end dates (i.e. they indicated a menses start date that occurred after date of survey completion). Participants were then categorized into one of four groups, Menses (*n* = 143), Follicular (*n* = 150), Early-Luteal (*n* = 137), and Premenstrual (*n* = 173) based on the traditional phases of the menstrual cycle as described by Draper et al. [31]. Consideration was given to participants within the 29–35 day range of their menstrual cycle (*n* = 61), since normal menstrual cycle length is widely considered to be between 21 and 35 days [17, 18]. These participants were placed into the Premenstrual group. In addition, women who indicated that they started their period on the same day of survey completion (*n* = 2), were placed in the Menses group. A chi-square analysis revealed no significant differences between the four groups of participants on any categorical variable.

## Results

### Preliminary analyses

Statistics were conducted using IBM SPSS Version 28. Mean age was 19.1 years (*SD* = 1.6), and average BMI was 23.8 kg/m2 (*SD* = 5.2). There were no significant differences between the groups on these two continuous variables. The sample was demographically diverse, with 53.7% identifying as White, 29.1% as Hispanic or Latino, 15.1% as Black, 9.3% as Asian or Pacific Islander, and 5.3% as other. Furthermore, no participants reported being pregnant at the time of data collection. While 55.9% of the sample reported they were not using any form of contraception, others reported using the pill (37.6%), IUD (3.2%), shot (0.7%), implant (2.3%), and patch (0.3%). No participants reported being treated for amenorrhea, 4.8% of the sample reported being treated for PMS, and 0.8% reported being treated for dysmenorrhea.

Analyses were conducted to demonstrate the usefulness of the PROMIS-GI scale in discerning DGBI diagnoses. At the time of data collection, 5.3% of participants were receiving medical treatment for ulcerative colitis, IBS, gastritis, Crohn’s Disease, stomach ulcers, and/or IBD. The other 94.7% did not report receiving medical treatment for any of the above DGBI. Objective 3 was assessed using a subsample of 36 participants with these confirmed diagnoses (placed in a DBGI group), and a matched randomized subsample of 32 participants with no self-reported medical diagnosis. Participants were matched on age, menstrual group, contraceptive use, and BMI. Ultimately, four participants in the DGBI group could not be matched due to extreme BMI and/or absence of a normal menstrual cycle and were therefore not included in the subsample. The final subsample consisted of 32 participants with DGBI diagnoses and 32 participants without DGBI diagnoses (*n* = 64).

### Objectives 1 and 2

An initial MANCOVA was conducted on the seven PROMIS-GI scales with menstrual group as the independent variable and health anxiety score as a covariate. No significant main effect for menstrual group was obtained, indicating that the PROMIS-GI measures did not significantly differ between the four menstrual groups. A significant main effect was obtained for health anxiety, *F*(7, 591) = 22.73, *p* < 0.001, *η*_*p*_^*2*^ = 0.21. Health anxiety scores had a significant effect on each PROMIS-GI score including belly pain, constipation, diarrhea, gas, nausea, swallowing, and reflux. There was no significant interaction between menstrual group and health anxiety ratings.

An additional MANCOVA examined the nine areas of the Pain Map across the four menstrual groups while controlling for health anxiety. A significant multivariate effect was obtained for SHAI score, *F*(9, 589) = 6.84,* p* < 0.001, *η*_*p*_^*2*^ = 0.10, indicating a significant relationship between health anxiety and the nine reported pain areas across all menstrual groups. Region H (Hypogastric Region) on the Pain Map significantly differed between menstrual groups, *F*(3, 48.29) = 5.36, *p* < 0.001, *η*_*p*_^*2*^ = 0.03. The Menses group (*M* = 4.37, *SD* = 3.14) reported more pain in Region H (Hypogastric Region) than did the Early-Luteal group (*M* = 2.91, *SD* = 3.03) and the Premenstrual group (*M* = 3.08, *SD* = 3.15). Ultimately, pain ratings in this region did not significantly differ between the Follicular phase and any other phase of the menstrual cycle. Early-Luteal and Premenstrual groups also did not significantly differ in Region H (Hypogastric Region) pain ratings.

### Objective 3

A MANOVA was conducted with the DGBI group and their matched controls as the predictor and PROMIS-GI total scores as outcome variables. A significant multivariate main effect for DGBI group was found, Pillai’s Trace = 0.312, F (7, 56) = 3.63, *p* = 0.003, *η*_*p*_^*2*^ = 0.31. Between–subjects effects for DGBI group by PROMIS-GI total scores are reported in Table 1.

**Table 1:** Between-subjects comparisons of PROMIS-GI T-Scores by DGBI diagnosis

PROMIS-GI Scale	DGBI Diagnosis				F (1,62)	p	*η*_*p*_^*2*^
	Yes (*n*=32)		No (*n*=32)				
	M	SD	M	SD			
Belly Pain	61.83	7.75	52.73	10.11	16.30	<.001	.21
Constipation	55.02	6.98	50.75	7.92	5.22	.026	.08
Gas	59.64	6.22	54.68	6.09	10.37	.002	.143
Reflux	49.64	7.94	44.16	6.94	8.66	.005	.123

Higher t-scores on PROMIS-GI measures indicate higher symptom severity

A second MANOVA was conducted with DGBI group as the predictor and scores for each quadrant of the Pain Map as outcome variables. There was a significant between-subjects effect for Regions B (Epigastric), E (Umbilical), G (Right Iliac), H (Hypogastric), and I (Left Iliac) of the Pain Map. Full between-subjects effect for DGBI group by Pain Map quadrants can be viewed in Table 2. Overall, those with a formal DGBI diagnosis reported significantly more pain in those regions than those without a DGBI diagnosis. These results underscore the effectiveness of the PROMIS-GI at tracking DGBI symptoms, even though the measures are not sensitive to variations of the menstrual cycle.

**Table 2:** Between-Subjects Comparisons of Pain Map Region by DGBI diagnosis

Pain Map Region	DGBI Diagnosis				F (1,62)	p	*η*_*p*_^*2*^
	Yes (*n* = 32)		No (*n* = 32)				
	M	SD	M	SD			
Region A	1.31	2.38	.50	1.16	3.02	.087	.046
Region B	1.94	2.80	.63	1.34	5.71	.020*	.084
Region C	1.09	2.20	.65	1.31	.96	.330	.015
Region D	1.34	2.03	1.16	1.78	.16	.695	.002
Region E	4.50	2.66	2.38	2.39	11.50	.001**	.156
Region F	1.66	2.28	1.25	2.00	.57	.452	.009
Region G	2.97	2.99	1.47	2.14	5.33	.024*	.079
Region H	5.34	2.60	2.91	2.75	13.28	< .001**	.176
Region I	3.19	3.02	1.44	2.37	6.65	.012*	.097

Higher scores on Pain Map regions indicate higher pain ratings on a 0 to 10 point scale

^*^ < .05

^**^ < .01

## Discussion

Evidence from many sources suggest that women experience higher levels of GI symptoms compared to men. Few studies have examined the role of the menstrual cycle as a reason for this difference in reported GI symptoms. This study examined the relationship between higher levels of GI symptoms reported by women across different phases of the menstrual cycle. The PROMIS-GI Scales were used to assess the broad range of GI functioning and a Pain Map was used to identify regions of the abdomen where pain was experienced.

Overall, results indicated negligible variability in GI symptoms across the menstrual cycle. However, significant differences were observed regarding abdominal pain in the hypogastric area of the abdomen. Specifically, the Menses group reported higher levels of abdominal pain in the hypogastric region (Region H) compared to the Early-Luteal and Premenstrual groups. The hypogastric region is associated with the female reproductive organs that are most often implicated in menstrual pain. Taken together, the group difference in pain ratings of the hypogastric abdominal region is likely to originate from menses rather than GI symptoms due to its location and periodicity.

Health anxiety, on the other hand, showed significant covariance with all GI symptoms as well as all areas of the Pain Map. This is consistent with the literature that finds strong associations between GI symptoms and health anxiety. Since health anxiety is higher in women on a population level, it may be a driving factor for previously documented sex differences in GI symptom reporting [24]. Our results also provide evidence that health anxiety is relatively unchanged across menstrual phases, further strengthening its relationship with GI symptoms. An important clinical implication is that individuals with high levels of health anxiety and GI symptoms may benefit from referral to mental health providers who specialize in a relatively new area, “Psychogastroenterology” [43].

Given that the PROMIS GI Abdominal Pain scale does not provide specific location information, concurrent use of the Pain Map is encouraged to localize the regions of abdominal pain and to distinguish between menstrual-related pain and GI pain. The inclusion of the Pain Map in assessing abdominal pain is particularly important for diagnostic accuracy, to avoid significant diagnostic delay for serious (i.e., non-GI-related) conditions such as endometriosis [44]. Among the factors associated with diagnostic delays is the belief that women are not reliable in distinguishing between pelvic and abdominal pain [44, 45]. Our findings provide preliminary evidence that women are accurate in distinguishing regions of pain related to GI and menstruation. Providing visual tools helps document pain localization during assessment and treatment.

The PROMIS-GI scale was effective in discerning participants with formal medical DGBI diagnoses and those without formal medical DGBI diagnoses. The DGBI group reported significantly higher belly pain, constipation, gas, and reflux than the non-diagnosed DGBI group. In addition, participants in the DGBI group had significantly higher rating of pain in Quadrants B (Epigastric Region), E (Umbilical Region), G (Right Iliac Region), H (Hypogastric Region), and I (Left Iliac Region) of the Pain Map. Thus, the Pain Map facilitated assessment of menstrual cycle pain and DGBI pain.

A previous study conducted by our lab with a different sample revealed that approximately a third of emerging adults (male and female) reported experiencing mild to severe GI symptoms [33]. Now, several years later in an exclusively female sample, we found that more than half of emerging adult women presented with GI symptoms whose severity is greater than normative levels. Evidently, young women continue to report elevated levels of GI symptoms, despite being otherwise healthy. Table 3 includes frequencies of GI symptom severity across PROMIS-GI measures in our latest sample.

**Table 3:** Frequency of GI Symptom Severity Ratings Across PROMIS-GI Scales

GI Symptom Severity Level	Belly Pain n (%)	Constipation n (%)	Diarrhea n (%)	Gas n (%)	Nausea n (%)	Swallow n (%)	Reflux n (%)
Normal	323 (53.6)	451 (74.8)	505 (83.7)	237 (39.3)	346 (57.4)	516 (85.6)	546 (90.5)
Mild	107 (17.7)	97 (16.1)	69 (11.4)	235 (39.0)	126 (20.9)	70 (11.6)	42 (7.0)
Moderate	157 (26.0)	55 (9.1)	28 (4.6)	126 (20.9)	129 (21.4)	17 (2.8)	15 (2.5)
Severe	16 (2.7)	––	1 (.2)	5 (.8)	2 (.3)	––	––

*N* = 603

GI symptom severity levels used the suggested PROMIS T-Score range of mild (T-scores between 55 and 60), moderate (T-scores between 60 and 70), and severe (T-scores above 70). There were no ratings that indicated Severe level of symptoms for either the Constipation, Swallow, or Reflux scales

Dysmenorrhea is a condition thought to affect between 45 and 90% of menstruating women and is characterized by painful menstruation accompanied by symptoms including nausea, diarrhea, vomiting, lower back pain, fatigue, and headache [46]. The rate of GI symptoms in our menses group is lower relative to studies that have focused on dysmenorrhea. Furthermore, only two women in our sample reported receiving a formal medical diagnosis of dysmenorrhea. Chen and colleagues [47, 48] categorized women with dysmenorrhea into 3 distinct latent classes and found that an estimated 19–25% of women with self-reported dysmenorrhea experienced “multiple severe symptoms,” which included GI symptoms along with abdominal cramps, abdominal pain, lower back pain, and headaches. Given this information, it is possible that the participants in our menses group predominantly fall outside of the identified “multiple severe symptoms” phenotype. This could account for the heightened pain localized to the hypogastric region, without the parallel increase in GI symptoms one might expect. This underscores the heterogeneous nature of dysmenorrhea experiences and emphasizes the necessity for continued research in this population.

There were several limitations of this study. One such limitation was the classification of menstrual groups was based on participant self-report of start- and end-dates of most recent menstruation. Previous studies that include the day of ovulation generally confirm responses using laboratory testing (body temperature or hormone-level testing). We were unable to include these laboratory tests in our study due to its online nature, budgeting constraints, and the large number of participants. Self-report is always susceptible to participant recall error and could lead to error in group assignment. Although biobehavioral research recommendations have been discussed to improve accuracy of menstrual phase identification and consistency in menstrual health research, it has also been noted that the self-report of onset of menses method is often the only feasible option. Importantly, our results likely contain some error in menstrual phase grouping, and particularly misclassification in the early-follicular and premenstrual groups [49]. The utilization of a 7-day recall period for PROMIS-GI assessments may have further limited the ability to detect subtle variations in GI symptoms across menstrual cycle phases and contributed to a lack of differences in scores between groups. Previous research varying the recall period for PROMIS physical scales has shown minimal effects on interpretations [50]. Nonetheless, these temporal constraints should be carefully considered when interpreting results of this study. Further research should also explore these issues with urinary LH testing and sex hormone measurement via saliva and/or blood sample, if possible.

Another limitation was the assumption of a 28–35-day menstrual cycle. Women who reported a period length greater than 35 days were excluded from the study. Thus, findings from this study may only be generalized to women who report a regular 28–35-day cycle. Since irregular period may be an indicator of other health abnormalities, including but not limited to GI diseases [33, 51], future studies should include women who do not report a regular menstrual cycle length. Those with irregular menstrual cycles may present differently than what we found in this study. Furthermore, we did not exclude females with other menstrual cycle irregularities such as anovulation. It has been established that college-aged females are at an increased risk of stress and disordered eating that can lead to menstrual irregularities, and future studies should aim to control for these factors. This study employed a cross-sectional design. Indeed, longitudinal studies will be essential to examine the long-term relationship between menstrual cycle phases and GI symptom patterns at the population level. Overcoming challenges such as individual variation in menstrual cycle timing and ensuring a sufficiently large sample size will be imperative for establishing robust conclusions. Finally, our sample consisted of women between the ages of 18–25. There is a need to replicate this study with a wider age range to identify the effects of the menstrual cycle on GI symptoms in premenopausal women of all ages.

## Conclusions

Menstrual cycle phase was not associated with differences in GI symptom severity on the PROMIS-GI scales, however we did find a characteristic difference in abdominal pain for women in the Menses phase. While our findings are most likely consistent with the everyday practice and observations of experienced clinicians, this study provides the first investigation into using standardized measures to quantify a full range of GI symptoms across the phases of the menstrual cycle. GI symptoms during all phases were highly impacted by health anxiety, which could possibly explain the observed sex-based differences in GI pain. Results from this study provide a simple approach to distinguish between GI- and menstrual-related abdominal pain and highlight the importance of routinely using tools such as a visual pain map during assessment and diagnosis. Based on our findings, a case can be made to collect PROMIS-GI measures, pain maps, and questions about the menstrual cycle in patient care settings, not only to facilitate GI symptom tracking, but also to differentiate features of the menstrual cycle from potential DGBI.

## Data Availability

The dataset used and analyzed during the current study is available from the corresponding author on reasonable request.

## Abbreviations

DGBI: Disorder of the Gut-Brain Interaction
FGID: Functional Gastrointestinal Disorder
IBS: Irritable Bowel Syndrome
GI: Gastrointestinal
IBD: Inflammatory Bowel Disease
GERD: Gastroesophageal Reflux Disorder
PSS: Perceived Stress Scale
SHAI: Short Health Anxiety Inventory
PANAS: Positive and Negative Affect Schedule
PROMIS-GI: Patient Reported Outcomes Measurement Information System- Gastrointestinal
NIH: National Institute of Health
GER: Gastroesophageal Reflux
IBM SPSS: International Business Machines Statistical Package for Social Sciences
BMI: Body Mass Index
MANCOVA: Multivariate Analysis of Covariance
